# Blockade of Electron Transport at the Onset of Reperfusion Decreases Cardiac Injury in Aged Hearts by Protecting the Inner Mitochondrial Membrane

**DOI:** 10.1155/2012/753949

**Published:** 2012-04-23

**Authors:** Qun Chen, Thomas Ross, Ying Hu, Edward J. Lesnefsky

**Affiliations:** ^1^Pauley Heart Center, Division of Cardiology, Department of Internal Medicine, Virginia Commonwealth University, Richmond, VA 23298, USA; ^2^Department of Biochemistry, Virginia Commonwealth University, Richmond, VA 23298, USA; ^3^Cardiology Section, Medical Service 111 (J), McGuire VA Medical Center, Richmond, VA 23249, USA

## Abstract

Myocardial injury is increased in the aged heart following ischemia-reperfusion (ISC-REP) compared to adult hearts. Intervention at REP with ischemic postconditioning decreases injury in the adult heart by attenuating mitochondrial driven cell injury. Unfortunately, postconditioning is ineffective in aged hearts. Blockade of electron transport at the onset of REP with the reversible inhibitor amobarbital (AMO) decreases injury in adult hearts. We tested if AMO treatment at REP protects the aged heart via preservation of mitochondrial integrity. Buffer-perfused elderly Fischer 344 24 mo. rat hearts underwent 25 min global ISC and 30 min REP. AMO (2.5 mM) or vehicle was given for 3 min at the onset of REP. Subsarcolemmal (SSM) and interfibrillar (IFM) mitochondria were isolated after REP. Oxidative phosphorylation (OXPHOS) and mitochondrial inner membrane potential were measured. AMO treatment at REP decreased cardiac injury. Compared to untreated ISC-REP, AMO improved inner membrane potential in SSM and IFM during REP, indicating preserved inner membrane integrity. Thus, direct pharmacologic modulation of electron transport at REP protects mitochondria and decreases cardiac injury in the aged heart, even when signaling-induced pathways of postconditioning that are upstream of mitochondria are ineffective.

## 1. Introduction

The aged heart sustains increased injury during ischemia-reperfusion in both experimental models [1–4] and in elderly patients [5]. Aging hearts are also resistant to the powerful endogenous protections provided by ischemic preconditioning and postconditioning [6–12]. Pharmacological conditioning such as anesthetic preconditioning also does not protect the aging heart during ischemia-reperfusion [13]. Aging causes dysfunction in interfibrillar mitochondria [14]. The aging defect in complex III and cytochrome oxidase decreases oxidative phosphorylation and increases the generation of reactive oxygen species [14–16]. Improvement of age-induced mitochondrial dysfunction with supplementation of acetylcarnitine decreases myocardial injury during ischemia-reperfusion [17], supporting that the electron transport chain defects present in the aged heart contribute to the increased myocardial injury.

Cardiac ischemia damages the electron transport chain and leads to the increased generation of reactive oxygen species (ROS) and mitochondrial calcium over load [18–22]. Opening of the mitochondrial permeability transition pore (MPTP) is a critical step in the onset of cardiomyocyte death [23, 24]. The increased generation of ROS and calcium overload are key factors that induce MPTP opening during cellular stress [25–28]. Although the increased production of ROS and calcium overload occur during ischemia [18], MPTP opening occurs mostly during reperfusion. Ischemia induces intracellular acidification that blunts MPTP opening [25–28]. Ischemic postconditioning decreases cardiac injury and prevents MPTP opening in adult hearts [21], supporting that MPTP mainly opens during reperfusion. Postconditioning leads to activation of cytoprotective signaling cascades, including the reperfusion injury salvage kinase pathway cascade (RISK) [29] and the tumor necrosis factor-STAT3 cascade (SAFE) [30]. Intervention during early reperfusion is still able to reduce cardiac injury although ischemic damage to the electron transport chain has already occurred [21, 31]. In fact, in the adult heart, postconditioning appears to work via protection against MPTP in mitochondria that have sustained damage from ischemia [32]. Unfortunately, ischemic postconditioning does not protect aged myocardium [6, 33, 34]. Alternative approaches must be found to decrease cardiac injury in the aged heart.

Blockade of electron transport with amobarbital during ischemia protects cardiac mitochondria and decreases myocardial injury in adult hearts [18, 19, 35]. Amobarbital given before ischemia also decreases cardiac injury in aged rat hearts measured after reperfusion [36]. These findings suggest that direct manipulation of mitochondrial function is an alternative approach to protect aged hearts that lack effective endogenous cytoprotective mechanisms.

Although prevention of mitochondrial damage during ischemia by amobarbital treatment [18, 19, 35] or ischemic preconditioning [6–8, 34] is an optimal strategy for cardioprotection, the clinical relevance of these treatments is limited due to the unpredictable occurrence of ischemic events. Interventions applied at the onset of reperfusion are practical for therapeutic use in the treatment of acute myocardial infarction [21, 37]. Direct blockade of electron transport by amobarbital at the onset of reperfusion decreases myocardial injury in the adult heart [19, 31]. In the present study, we tested if blockade of electron transport at the onset of reperfusion was able to decrease cardiac injury in the aged heart. We also evaluated if protection by direct modulation of mitochondrial function during early reperfusion is mediated by the protection of mitochondrial inner membrane integrity.

## 2. Methods

### 2.1. Preparation of Rat Hearts for Perfusion

 The Animal Care and Use Committees of the McGuire VA Medical Center and Virginia Commonwealth University approved the protocol. Male Fischer rats (24 mo.) were anesthetized with pentobarbital sodium (100 mg/kg i.p.) and anticoagulated with heparin (1000 IU/kg i.p.). Hearts were excised and perfused retrograde via the aorta in the Langendorff mode with modified Krebs-Henseleit (K-H) buffer oxygenated with 95% O_2_/5% CO_2_ as previously described [35]. Cardiac function was monitored with a balloon inserted into the left ventricle.

### 2.2. Protocol for Heart Ischemia and Reperfusion and Amobarbital Perfusion

**Figure 1:**
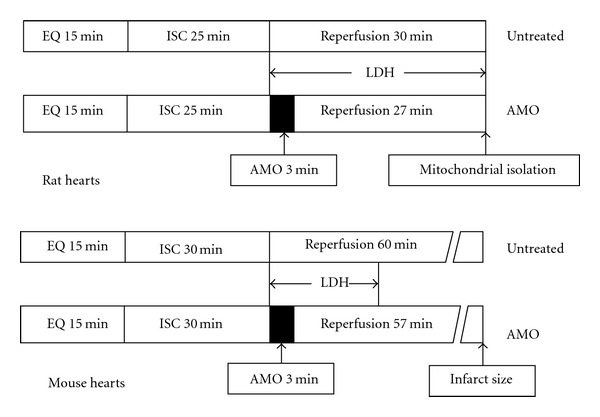
Time course of ischemia and reperfusion. In the untreated rat heart group after 25 min global ischemia, the heart underwent 30 min reperfusion. In the amobarbital- (AMO-) treated group, the drug (2.5 mM) was given for three min at the onset of reperfusion followed by 27 min of untreated Krebs-Henseleit buffer perfusion (upper panel). In isolated mouse hearts, hearts were subjected to 30 min global ischemia and 60 min reperfusion with and without amobarbital treatment. The infarct size was determined at the end of reperfusion (lower panel).

 In untreated hearts, the heart was perfused for 15 min with K-H buffer, followed by 25 min global ischemia at 37°C and 30 min reperfusion. In the amobarbital treated group, hearts followed the same perfusion protocol except that amobarbital (2.5 mM) was perfused in identical K-H buffer for three min at onset of reperfusion (Figure 1, upper panel) [35]. Hearts were paced at 300 beats per min during the 15 min equilibration period and again after 10 min reperfusion. Coronary effluent was collected during the 30 min reperfusion period, and LDH activity in coronary effluent was determined to reflect myocardial injury [35].

### 2.3. Determination of Myocardial Infarct Size

 In order to further evaluate the amobarbital-mediated cardiac protection, myocardial infarct size was measured in buffer-perfused mouse hearts in the absence and presence of amobarbital treatment at the onset of reperfusion. In untreated hearts, the heart was perfused for 15 min with K-H buffer, followed by 30 min global ischemia at 37°C and 60 min reperfusion [38]. In the amobarbital-treated group, hearts followed the same perfusion protocol except that amobarbital was perfused in identical K-H buffer for three min at onset of reperfusion (Figure 1, lower panel) [35]. Hearts were paced at 420 beats per min during the 15 min equilibration period and again after 10 min reperfusion. Coronary effluent was collected during the 30 min reperfusion period, and LDH activity in coronary effluent was determined to reflect myocardial injury [35]. Myocardial infarct size was determined using TTC staining [35].

### 2.4. Mitochondrial Isolation and Functional Assessment

Subsarcolemmal (SSM) and interfibrillar (IFM) mitochondria were isolated from hearts at the end of the experiment [35]. Oxidative phosphorylation was determined using a Clark-type oxygen electrode at 30°C [35]. H_2_O_2_ production from intact mitochondria was measured using the oxidation of the fluorogenic indicator Amplex Red in the presence of horseradish peroxidase (HRP) [39].

### 2.5. Measurement of Mitochondrial Inner Membrane Potential

Mitochondrial inner membrane potential was assessed using the fluorescence intensity of the indicator TMRM (tetramethylrhodamine, methyl ester) [40]. TMRM is a lipophilic cation accumulated by mitochondria in proportion to Δ*ψ* (emission at 590 nM, excitation at two wavelengths: 573 nm and 546 nm). Accumulated TMRM in mitochondria causes a fluorescence “red shift,” and fluorescence intensity was quantified by calculating the ratio of intensity from 573 to intensity from 546 (573/546). TMRM was selected to reflect membrane potential since TMRM did not inhibit oxidative phosphorylation at the concentration (0.3 *μ*M) used [40]. The Δ*ψ* was reflected by Δ change of fluorescence intensity of TMRM in the presence of ADP (2 mM), oligomycin (1 *μ*M), and DNP (0.2 mM/each) using glutamate (10 mM) as a complex I substrate (Figure 4).

### 2.6. Determination of Calcium Tolerance Capacity (CRC)

Mitochondrial tolerance to calcium loading was studied in the single-cell fluorometer using repetitive calcium pulses [21, 41]. Freshly isolated SSM and IFM (0.2 mg/mL) were incubated in buffer (150 mM sucrose, 50 mM KCl, 2 mM KPi, and 20 mM Tris/HCl, pH 7.4) for 90 sec. with stirring at 30°C with 0.5 *μ*M calcium green. Succinate (5 mM) was used as substrate. Pulses of calcium (20 nmoles) were added at 1 min intervals. The number of pulses that resulted in calcium release (MPTP opening) was used to calculate CRC (Figure 6(a)).

### 2.7. Statistical Analysis

Data are expressed as the mean ± standard error of the mean. Differences among groups were compared by two-tailed *t*-test (*SigmaStat 3.5*, ProgramPaketet, Gothenburg, Sweden). Two-way ANOVA was used to analyze the hemodynamic data and inner mitochondrial membrane potential, and post hoc test was used to show statistical difference between groups. A difference of *P* < 0.05 was considered significant.

## 3. Results

### 3.1. Reversible Blockade of Electron Transport at Onset of Reperfusion Decreased Myocardial Injury

**Figure 2:**
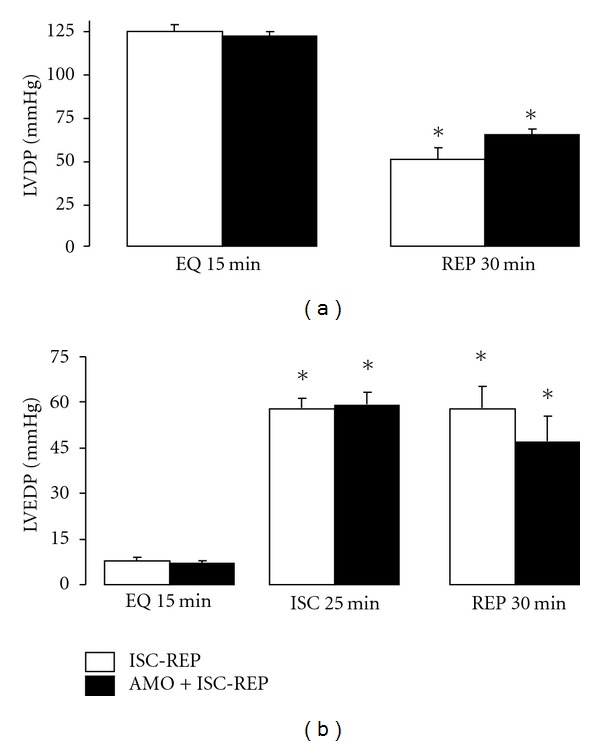
Hemodynamic changes during ischemia and reperfusion. There were no differences in left ventricular developed pressure (LVDP) and diastolic pressure (LVEDP) between the two groups at the end of the 15 min equilibration (EQ) period before ischemia. The diastolic pressure in untreated hearts was not different in the two groups at the end of ischemia, prior to treatment. LVDP and LVEDP were similar in both groups during reperfusion. (Mean ± SEM; *P < 0.05 versus preischemia value, *n* = 10 in each group.)

**Figure 3:**
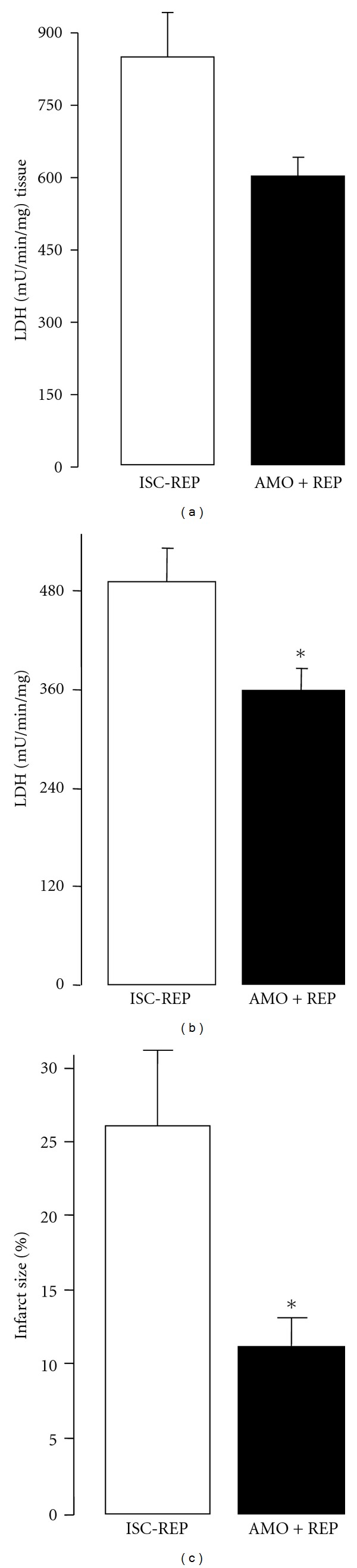
LDH release during reperfusion in isolated rat hearts (a). LDH activity in coronary effluent during 30 min reperfusion was measured in order to assess myocardial injury. Amobarbital given at the onset of reperfusion markedly decreased LDH release compared to untreated hearts, supporting that the modulation of mitochondrial respiration during early reperfusion decreased myocardial injury in the aged hearts. Amobarbital treatment also decreased LDH release in buffer perfused mouse hearts (b) and myocardial infarct size (c). (Mean ± SEM; *P < 0.05 versus untreated hearts *n* = 6–10 in each group.)

There were no differences in hemodynamic data (left ventricular developed pressure-LVDP and left ventricular end diastolic pressure-LVEDP) between the two groups at the end of the equilibration period before ischemia (Figure 2). Ischemia markedly increased diastolic pressure (LVEDP) in both groups (Figure 2). There was no difference in LVEDP between the two groups at the end of ischemia, indicating that hearts in both groups suffered the same degree of ischemic contracture before treatment. The diastolic pressure remained elevated during reperfusion in untreated hearts. Amobarbital given during early reperfusion had minimal effect on diastolic pressure (Figure 2). In untreated hearts, LVDP is markedly decreased during reperfusion compared to the preischemic value. Amobarbital given during reperfusion tended to improve the recovery of LVDP, but it did not reach statistical significance (Figure 2). In contrast to cardiac function, amobarbital given at the onset of reperfusion markedly decreased LDH release into coronary effluent compared to untreated hearts, indicating that modulation of electron transport during early reperfusion provided solid cardioprotection in aged hearts following reperfusion (Figure 3(a)). Amobarbital given during early reperfusion also protected buffer-perfused mouse hearts as shown by decreased LDH release (Figure 3(b)) and myocardial infarct size (Figure 3(c)). These data further support that reversible blockade of electron transport during early reperfusion decreased myocardial injury.

### 3.2. Reversible Blockade of Electron Transport at the Onset of Reperfusion Did Not Improve Mitochondrial Oxidative Phosphorylation

Amobarbital given during reperfusion did not improve oxidative phosphorylation in SSM or IFM (glutamate, succinate, and TMPD-ascorbate as complex I, II, and IV substrates, resp.) compared to untreated hearts (Table 1). Amobarbital treatment also did not alter uncoupled respiration induced by dinitrophenol (DNP) compared to untreated hearts (Table 1). Thus, manipulation of respiration during early reperfusion did not protect the electron transport chain. This result was consistent with our previous findings that the damage to the electron transport chain occurred during ischemia, rather than during reperfusion [21, 35, 42].

### 3.3. Reversible Blockade of Electron Transport at the Onset of Reperfusion Improved Mitochondrial Inner Membrane Potential

**Figure 4:**
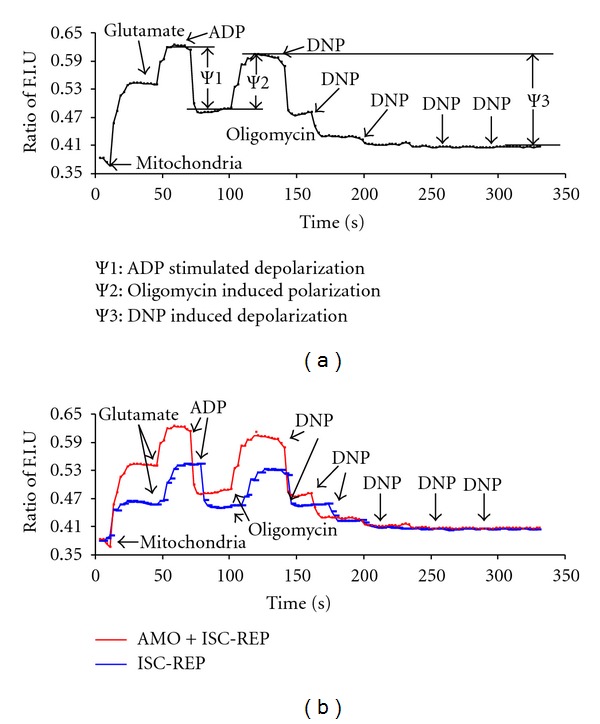
Measurement of mitochondrial inner membrane potential. The upper panel is an original tracing of an inner membrane potential measurement. Glutamate (10 mM) was used as a complex I substrate to polarize the inner membrane potential. ADP stimulated the depolarization of inner membrane potential. Inner membrane potential was restored when oligomycin was used to inhibit complex V. Dinitrophenol (DNP) was titrated to completely collapse inner membrane potential. The Δ change of fluorescence intensity was used to assess inner mitochondrial membrane potential in the presence of ADP, oligomycin, and DNP. The lower panel provides representative tracings of inner membrane potential from mitochondria from untreated (blue line) and amobarbital-treated (red line) hearts.

**Figure 5:**
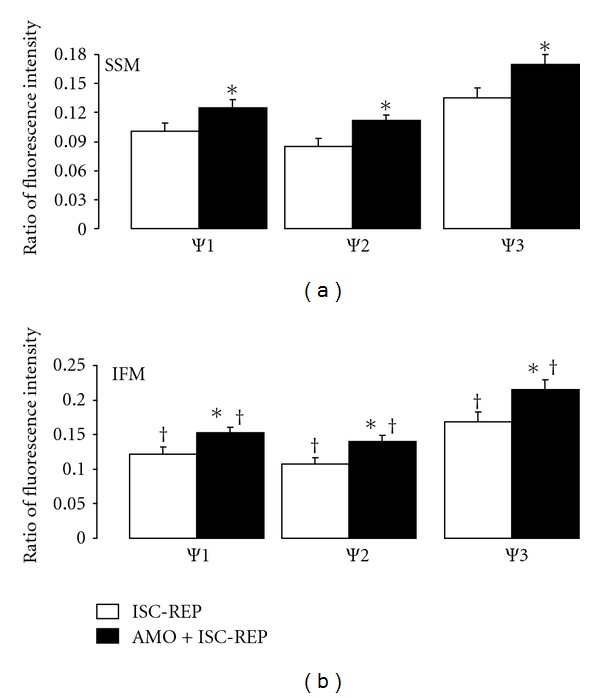
Mitochondrial inner membrane potential following ischemia-reperfusion. Amobarbital treatment during reperfusion improved inner mitochondrial potential measured following 30 minutes of reperfusion in both SSM (a) and IFM (b) in the presence of ADP (*ψ* 1), oligomycin (*ψ* 2), and DNP (*ψ* 3). *ψ* 1–3 conditions are as shown in Figure 4. (Mean ± SEM; *P < 0.05 versus untreated *n* = 10 in each group.)

Mitochondrial inner membrane potential was used to assess inner membrane permeability during reperfusion. An original tracing of inner membrane potential is shown in Figure 4. Mitochondrial inner membrane potential stimulated by ADP was significantly improved in both SSM and IFM by amobarbital treatment compared to untreated hearts (Figure 5). Amobarbital treatment improved inner membrane potential in the presence of oligomycin (complex V inhibitor) in both SSM and IFM, suggesting that complex V was not the site of the defect (Figure 5). Titration of DNP caused complete depolarization of mitochondria (Figure 4). Amobarbital treatment improved inner membrane potential with DNP titration in both SSM and IFM (Figure 5).

### 3.4. Reversible Blockade of Electron Transport at Onset of Reperfusion Did Not Improve Mitochondrial Calcium Retention Capacity (CRC)

**Figure 6:**
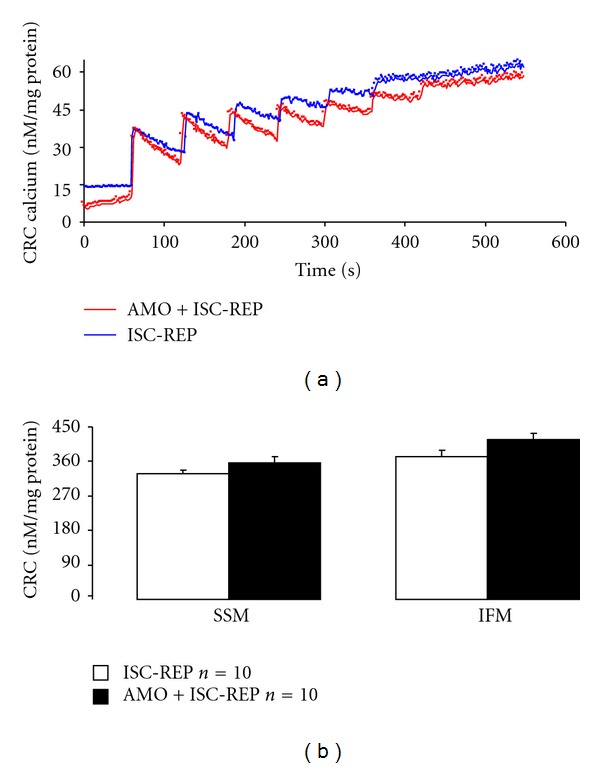
Measurement of mitochondrial calcium tolerance. Succinate (10 mM) was used to energize mitochondria. The upper panel (a) is an original tracing of calcium retention capacity (CRC). A calcium pulse (20 nmoles) was added each time. The release of calcium from mitochondria reflected opening of the permeability transition pore. The lower panel (b) shows that amobarbital treatment during reperfusion did not improve calcium tolerance in SSM and IFM compared to mitochondria from untreated hearts. (Mean ± SEM. *P* = NS versus untreated *n* = 10 in each group.)

CRC was used to assess MPTP opening in isolated mitochondria [21]. An original tracing of CRC measurement is shown in Figure 6(a). Amobarbital treatment during reperfusion did not significantly improve CRC in SSM and IFM compared to untreated hearts (Figure 6(b)).

### 3.5. Blockade of Electron Transport at the Onset of Reperfusion Decreases H_2_O_2_ Generation

**Figure 7:**
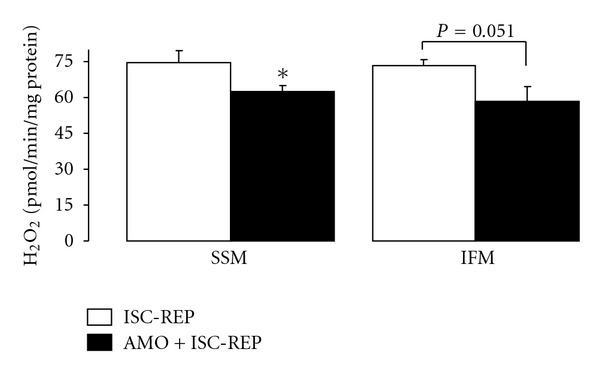
The generation of reactive oxygen species in mitochondria following ischemia-reperfusion. Amobarbital treatment during early reperfusion decreased net H_2_O_2_ production in subsarcolemmal mitochondria (SSM) compared to untreated hearts when glutamate was used as a complex I substrate. Amobarbital also tended to decrease net H_2_O_2_ production in interfibrillar mitochondria (IFM) compared to untreated hearts (*P* < 0.06). Data are expressed as mean ± SEM; *P < 0.05 versus untreated. *N* = 5 in each group.

Mitochondrial electron transport is a major source of ROS generation [22, 43]. Using glutamate as a complex I substrate, amobarbital treatment decreased the generation of H_2_O_2_ in SSM compared to untreated hearts (Figure 7). The amount of H_2_O_2_ generated from IFM from amobarbital treated hearts also was lower than that in the untreated group (*P* = 0.051). There was no difference in H_2_O_2_ production between the two groups in both SSM and IFM using succinate + rotenone as a complex II substrate (H_2_O_2_ pmol/mg/min Mean ± SEM: SSM,  109 ± 5  untreated versus  96 ± 9  amobarbital; IFM,  111 ± 3  untreated versus  114 ± 8  amobarbital, *P* = NS, *n* = 5 in each group).

## 4. Discussion

The novel findings of the present study are (1) reversible blockade of electron transport at the onset of reperfusion decreases myocardial injury in aged hearts; (2) the protection by amobarbital treatment during reperfusion was mediated by protection of the inner mitochondrial membrane. The present study indicates that modulation of mitochondrial respiration at the onset of reperfusion is able to decrease cardiac injury in the aged heart even when oxidative phosphorylation was already compromised from ischemia [42]. Thus, the aged heart can be protected in a manner similar to the adult heart by using this translationally relevant pharmacologic strategy to attenuate cardiac injury during reperfusion.

Aging leads to impaired mitochondrial function that augments myocardial injury during ischemia-reperfusion [15, 17, 42]. The loss of endogenous protective mechanisms in the aged heart provides a greater challenge to protect the aged heart during acute myocardial infarction and its treatment. The endogenous protective mechanisms can be restored in aging hearts by treatments such as caloric restriction [44], exercise [45], and a pharmacologic strategy to inhibit protein phosphatase 2A activity [46]. Supplementation of acetylcarnitine before ischemia decreases myocardial injury in aged hearts via restoration of mitochondrial respiration to an adult-phenotype [17]. However, these approaches must all be instituted before ischemia and require variable pretreatment periods in order to be effective. Due to the unpredictable occurrence of acute ischemic events, these strategies are unfortunately less clinically relevant to protect the ischemic-reperfused aged heart. In the present study, amobarbital given at the onset of reperfusion decreased myocardial injury. Compared to the pre-ischemia strategies, amobarbital treatment at reperfusion has therapeutic potential since amobarbital could be regionally infused during primary stent-mediated reperfusion treatment of ST elevation acute infarcts in the high risk elderly patient population [5].

 Amobarbital given before ischemia decreases cell injury in isolated rabbit [19], rat [35, 47], and guinea pig hearts [18]. Amobarbital given before ischemia prevents electron transport chain damage measured following reperfusion in isolated adult [35] and aged rat hearts [36]. In the present study, amobarbital given at the onset of reperfusion does not protect the electron transport chain in aged hearts. Taken together, these results further support the notion that the damage of mitochondrial electron transport chain mainly occurs during ischemia, rather than during reperfusion [48], including in the aged heart [49]. In the adult heart, amobarbital treatment during reperfusion decreases cardiac injury without improving oxidative phosphorylation [31] indicating that the mechanism of myocyte death during reperfusion is not solely dependent on the function of mitochondrial respiration. Ischemic postconditioning, another well known method applied during early reperfusion, also fails to improve oxidative phosphorylation in the adult heart [21, 50]. Thus, manipulation of ischemia-damaged mitochondria either via activation of cytoprotective signaling systems in the adult heart [29, 50–52] or this novel direct manipulation of mitochondrial function in the aged heart is able to decrease myocardial injury during reperfusion.

MPTP opening contributes to myocyte death during ischemia-reperfusion [23, 24, 53]. Although the exact structure of the MPTP remains elusive, the effect of MPTP opening is clear: its opening leads to increased permeability of both mitochondrial inner and outer membranes [23, 26]. Mitochondrial inner membrane potential (Ψ) is commonly used to reflect the permeability of the inner membrane [25, 53], including from MPTP. In the present study, inner membrane potential was used to reflect inner membrane integrity in isolated mitochondria. Amobarbital treatment preserves inner membrane potential in both SSM and IFM compared to untreated hearts, suggesting that amobarbital decreases the permeability of inner membrane during reperfusion. In the presence of oligomycin [54], inner membrane potential remains lower in mitochondria from untreated hearts compared to amobarbital treatment, indicating that there is a consistent proton leakage site other than complex V. Ischemia-reperfusion may activate uncoupling proteins that allow proton backflow into the matrix [54]. If this was the case, we anticipate that the difference of Ψ between untreated and amobarbital-treated mitochondria would be eliminated in the presence of uncoupler, DNP. Amobarbital improves inner membrane potential in the presence of DNP, suggesting that uncoupling proteins are a less likely mechanism for the proton leak. Another potential proton leak site is the adenine nucleotide translocase (ANT) [54]. ANT was once considered a key component of the MPTP, but the role of ANT in the pore is now uncertain [55]. We found that inhibition of ANT using bongkrekic acid did not restore inner membrane potential in mitochondria following ischemia-reperfusion (data not shown). These results indicate that the impairment of inner membrane potential in mitochondria isolated following ischemia-reperfusion in the aged heart is not through the specific sites discussed above.

The improved inner membrane potential following amobarbital treatment suggests that treatment decreases MPTP opening during reperfusion. However, amobarbital treatment does not improve mitochondrial calcium tolerance (calcium retention capacity—CRC), another common index used to reflect MPTP [21, 56]. These results indicate that the mechanism by which amobarbital treatment decreases inner mitochondrial membrane permeability is not solely through prevention of MPTP opening. Cardiolipin is a unique phospholipid located in the inner membrane [48, 57, 58], and insufficient cardiolipin content or the presence of oxidized cardiolipin increases inner membrane permeability [59, 60]. Aging does not alter cardiolipin content [61] but results in the enhanced formation of oxidized cardiolipin species in both SSM and IFM following ischemia compared to adult hearts [49]. The increases in oxidized cardiolipin content persist during reperfusion in the aged heart [49], suggesting ongoing production during the oxidative stress of reperfusion. Amobarbital treatment during reperfusion may decrease the permeability of the inner membrane by attenuating the ongoing production of oxidized cardiolipin during reperfusion. This concept is an area of ongoing study in our laboratory.

The burst of ROS formation at the onset of reperfusion contributes to myocardial injury [22]. The ischemia-damaged electron transport chain increases the generation of ROS during reoxygenation in isolated mitochondria [62]. Protection of mitochondrial electron transport during ischemia decreases ROS generation during reperfusion [35, 36]. In the present study, amobarbital given during early reperfusion decreases ROS generation from isolated mitochondria obtained from the aged heart, suggesting that amobarbital decreases myocardial injury by decreasing ROS formation during reperfusion, perhaps leading to decreased production of oxidized cardiolipin and thereby protecting the inner mitochondrial membrane. Ischemia increases ROS generation from complex I and complex III [62]. In the present study, amobarbital treatment at the onset of reperfusion was likely protective by two complimentary mechanisms. First, amobarbital decreased electron flow into complex III, which produces increased oxidative injury from both age-related [14–16] and ischemia-induced [35] defects, the latter observed online in the intact heart [18]. Second, amobarbital treatment decreases complex-I-mediated complex I damage [19, 35] with decreased production of ROS from complex I as observed during reperfusion in the current study.

Reperfusion in clinical settings usually occurs following at least moderate periods of ischemia that result in mitochondrial damage. Transient interruption of mitochondrial oxidative metabolism only during early reperfusion decreases myocardial injury in aged hearts providing a relevant approach to limit myocardial cell death during reperfusion in the high-risk elderly population.

## Figures and Tables

**Table 1:** Oxidative phosphorylation in SSM and IFM following ischemia-reperfusion.

	SSM		IFM	
	ISC-REP	AMO + REP	ISC-REP	AMO + REP
Glutamate-ADP	94 ± 10	109 ± 11	123 ± 11	145 ± 14
Glutamate-DNP	93 ± 9	114 ± 13	121 ± 12	149 ± 15
Succinate-ADP	100 ± 9	124 ± 10	127 ± 10	154 ± 13
Succinate-DNP	97 ± 9	119 ± 10	121 ± 10	148 ± 13
TMPD-ADP	408 ± 23	438 ± 16	488 ± 28	560 ± 36

Data are expressed as mean ± SEM; *P* = NS, ISC-REP versus AMO + REP, *N* = 10 in each group.
